# CD4^+^CD25^+^FOXP3^+^ regulatory T cells: a potential “armor” to shield “transplanted allografts” in the war against ischemia reperfusion injury

**DOI:** 10.3389/fimmu.2023.1270300

**Published:** 2023-10-06

**Authors:** Shareni Jeyamogan, Joseph R. Leventhal, James M. Mathew, Zheng Jenny Zhang

**Affiliations:** ^1^Department of Surgery, Comprehensive Transplant Center Feinberg School of Medicine, Northwestern University, Chicago, IL, United States; ^2^Simpson Querrey Institute for BioNanotechnology, Feinberg School of Medicine, Northwestern University, Chicago, IL, United States; ^3^Department of Microbiology-Immunology, Feinberg School of Medicine, Northwestern University, Chicago, IL, United States; ^4^Microsurgery and Pre-Clinical Research Core, Comprehensive Transplant Center, Feinberg School of Medicine, Northwestern University, Chicago, IL, United States

**Keywords:** ischemia, reperfusion, regulatory T cells, anti-inflammation, oxidative stress, allografts, immune tolerance

## Abstract

Despite the advances in therapeutic interventions, solid organ transplantation (SOT) remains the “gold standard” treatment for patients with end-stage organ failure. Recently, vascularized composite allotransplantation (VCA) has reemerged as a feasible treatment option for patients with complex composite tissue defects. In both SOT and VCA, ischemia reperfusion injury (IRI) is inevitable and is a predominant factor that can adversely affect transplant outcome by potentiating early graft dysfunction and/or graft rejection. Restoration of oxygenated blood supply to an organ which was previously hypoxic or ischemic for a period of time triggers cellular oxidative stress, production of both, pro-inflammatory cytokines and chemokines, infiltration of innate immune cells and amplifies adaptive alloimmune responses in the affected allograft. Currently, Food and Drug Administration (FDA) approved drugs for the treatment of IRI are unavailable, therefore an efficacious therapeutic modality to prevent, reduce and/or alleviate allograft damages caused by IRI induced inflammation is warranted to achieve the best-possible transplant outcome among recipients. The tolerogenic capacity of CD4^+^CD25^+^FOXP3^+^ regulatory T cells (Tregs), have been extensively studied in the context of transplant rejection, autoimmunity, and cancer. It was not until recently that Tregs have been recognized as a potential cell therapeutic candidate to be exploited for the prevention and/or treatment of IRI, owing to their immunomodulatory potential. Tregs can mitigate cellular oxidative stress, produce anti-inflammatory cytokines, promote wound healing, and tissue repair and prevent the infiltration of pro-inflammatory immune cells in injured tissues. By using strategic approaches to increase the number of Tregs and to promote targeted delivery, the outcome of SOT and VCA can be improved. This review focuses on two sections: (a) the therapeutic potential of Tregs in preventing and mitigating IRI in the context of SOT and VCA and (b) novel strategies on how Tregs could be utilized for the prevention and/or treatment of IRI.

## 1 Introduction

The alarming rate at which end-stage organ failure cases are being reported has become a major public health concern, and its burden is expected to double yearly, in part due to the increase in ageing population (1–4). Despite the advances in therapeutic interventions, solid organ transplantation (SOT) is currently the “gold standard” treatment for patients with end-stage organ failure. Moreover, statistics from the Global Observatory on Donation and Transplantation (GODT) from World Health Organization (WHO), demonstrated that kidneys make up the majority of organ transplants followed by liver, heart, lung, pancreas (2, 3, 5) and the percentage of solid organ transplants are projected to increase yearly (2, 3, 5). Unlike SOT, vascularized composite allotransplantation (VCA) involves the transplantation of composite tissues comprising of multiple tissue types such as nerves, muscles, skin, bone, and blood vessels (6, 7). VCA, which is arguably more life-enhancing rather than a life-saving, recently has been deemed a reliable treatment option for patients with complex composite tissue injury (7). In SOT and VCA, besides alloantigen based immunogenicity, the occurrence of ischemia reperfusion injury (IRI) in transplanted allografts resulting in compromised allograft viability, function, and overall transplant outcome, remain a major challenge (8–10). IRI in transplantation, is a complex inflammatory phenomenon triggered by temporary deprivation of blood supply during donor organ procurement and exacerbated following, restoration of oxygenated blood supply after revascularization of the transplanted organ. In both SOT and VCA, IRI is inevitable and is a predominant factor for the onset of several life-threatening complications such as early graft dysfunction and/or graft rejection due to amplified, and prolonged alloantigen immunogenicity induced inflammatory activity (8–11).

To date, Food and Drug Administration (FDA) approved drugs for the treatment of IRI in the context of SOT and VCA are unavailable. Therefore, an efficacious approach or potential therapeutic modality to prevent, reduce and/or to alleviate allograft damages caused by IRI induced inflammation is warranted to achieve the best-possible transplant outcome among transplant recipients. Over the last few years, it has become evident that numerous myeloid lineage regulatory cells (e.g., Dendritic regulatory cells, polymorphonuclear myeloid suppressor cells) and lymphoid lineage regulatory cells (CD4^+^, CD8^+^ or B regulatory cells) play crucial roles in the maintenance of immune homeostasis. Among these regulatory cell populations, the discovery and identification of specific CD4^+^CD25^+^FOXP3^+^ regulatory T cells (Tregs) in both human and animal models, had led to unprecedented and extensive investigations on the therapeutic potential of these cells. Studies on autoimmune diseases and organ transplantation had demonstrated the ability of Tregs in inducing immunological tolerance and maintaining immune homeostasis by inhibiting and/or reducing the severity of pro-inflammatory events (12). Furthermore, the natural existence of Tregs in the immune system makes them an excellent candidate to be exploited for the treatment and/or prevention of numerous life-threatening immunological conditions and diseases.

The efficacy of Tregs in suppressing inflammatory responses driven by adaptive immune cells in SOT have been extensively studied and reviewed previously (12, 13). It is important to note that in addition to CD4+ Tregs, CD8+ Tregs are also able to induce immune tolerance and regulatory effects against inflammatory responses. However, due to their low frequency *in vivo*, the immunomodulatory potential of CD8^+^ Tregs against IRI have yet to be fully exploited (14). Due to the lack of studies on CD8^+^ Tregs, we decided to extensively review the therapeutic role of CD4^+^ Tregs in IRI induced inflammation. In this review, we first provide an overview of the pathophysiology of IRI in the transplant setting. We then review the therapeutic potential and possibilities of Tregs as immunomodulatory and anti-inflammatory mediators, in mitigating IRI induced inflammation. Lastly, we discuss the possible approaches and strategies which could be utilized to optimize the therapeutic efficacy of Tregs for the prevention and/or treatment of IRI in SOT and VCA.

## 2 Pathogenesis of ischemia and reperfusion injury in transplantation

It has been well established that the inflammatory responses in IRI are initiated during ischemia and are exacerbated during reperfusion (15). For instance, in kidney transplantation, prior to the removal of kidney from the donor, ischemia induced inflammation begins as soon as the donor’s blood supply to the kidney is stopped. During removal, the kidney graft undergoes a period of “warm ischemia” (9, 16) followed by, “cold ischemia” when the removed kidney is stored in a cold preservation solution to protect the viability of kidney cells (15, 17) and to slow down hypoxia mediated cellular stress and inflammatory responses exerted on the graft (18), for a period of time. Once the kidney is implanted into the recipient (before revascularization), the kidney graft experiences a short duration of “warm ischemia” (8–11, 16). Revascularization after implantation, enables the flow of oxygenated blood to the post-ischemic kidney graft (reperfusion), resulting in the activation and initiation of a cascade of pro-inflammatory events, acting interdependently on the affected kidney graft (16, 17). Depending on the severity of IRI, the harmful effects of IRI induced inflammatory responses, may promote graft rejection, graft dysfunction and/or even death (17). The unavoidable occurrence of IRI in both SOT and VCA warrants, a pressing need for alternative approaches and/or strategies to prevent, inhibit or at the very least to reduce IRI induced inflammatory responses against transplanted grafts.

### 2.1 Initiation of cellular stress during ischemia

**Figure 1:**
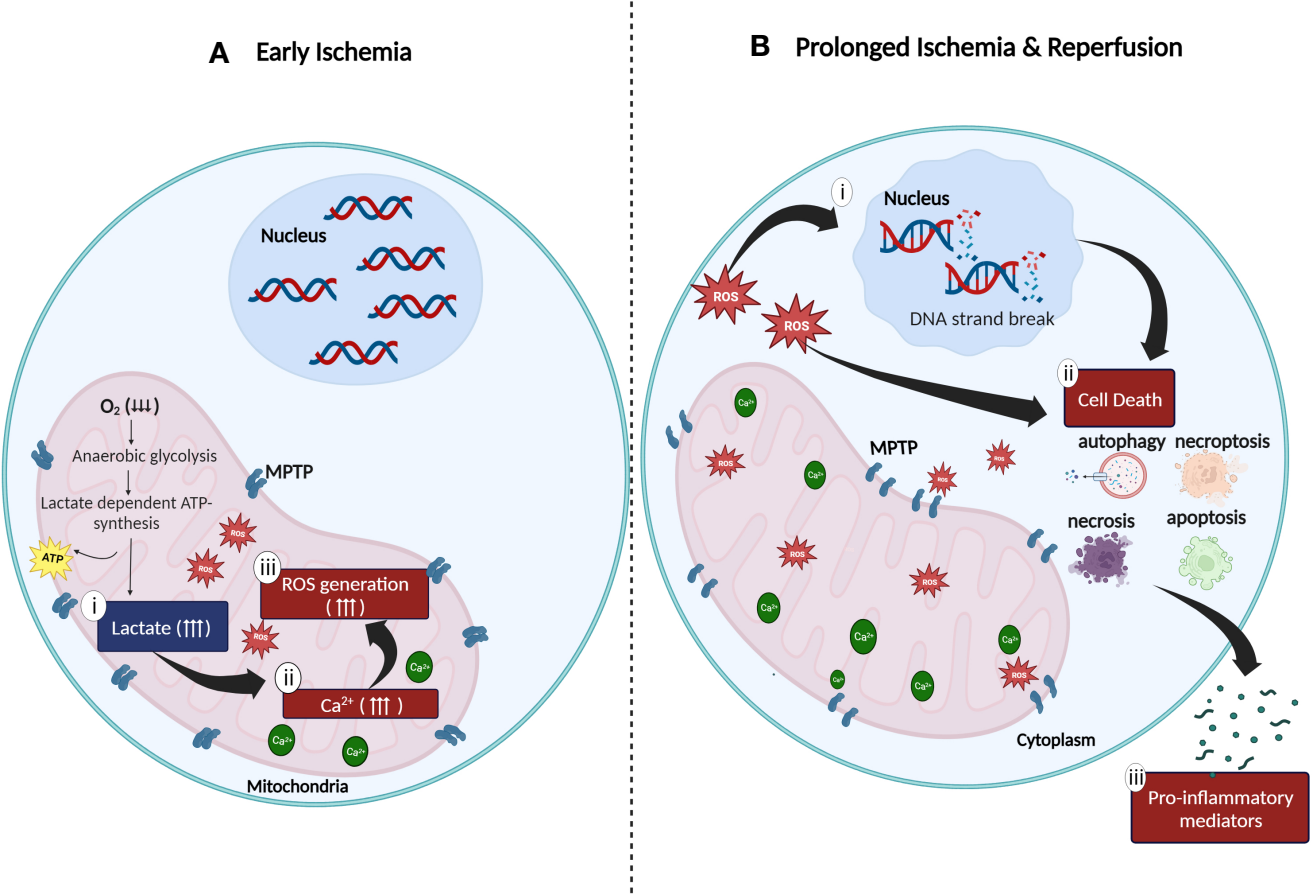
Cellular oxidative stress during **(A)** ischemia and **(B)** reperfusion injury. **(A)** Anaerobic metabolic activity during ischemia generates energy *via* the lactate dependent ATP pathway resulting in (i) lactate acidosis followed by (ii) accumulation of cytosolic calcium in the mitochondrial matrix due to compromised function of the sodium (Na^+^)-potassium (K^+^) pump. Increased concentration of mitochondrial Ca^2+^ generates (iii) high amount of ROS which is trapped in the mitochondria due to the closed mitochondrial permeability transition pores (MPTP). **(B)** Continuous stress on MPTP leads to the widening of MPTP which results in the release of ROS to the cytoplasm of cells. The release of ROS to the surrounding matrix leads to the (i) destruction of deoxyribonucleic acid (DNA) single strands and (ii) activation of cell death enzymes such as nuclear enzyme poly (ADP-ribose) synthetase which promotes apoptosis, necrosis, autophagy, and necroptosis. (iii) Excessive cell death mechanisms contribute to the release of pro-inflammatory mediators which further aggravates the inflammatory response (created with BioRender.com).

Oxygen is a critical component required in cellular metabolism and mitochondrial oxidative phosphorylation to produce energy. Hence, lack of oxygenated blood supply to tissues during hypoxia (ischemic period) impairs both, the mitochondrial energy production metabolic route and cellular metabolic activity (19, 20). The inhibition of mitochondrial oxidative phosphorylation activity caused by the absence of molecular oxygen directly impacts the generation of adenosine triphosphate (ATP) thereby, affecting glycolysis mechanism. As an alternate resort to produce ATP during hypoxia, the aerobic glycolytic pathway is switched to the anaerobic glycolytic pathway to ensure continuity of energy production (19). Consequently, since lactate (also known as lactic acid) is required for the production of ATP in anerobic glycolysis, mass production and accumulation of the extremely acidic lactate in the cytosolic region of mitochondria, compromises the efficacy of the mitochondrial sodium (Na^+^)-potassium (K^+^) pump function resulting in, increased mitochondrial membrane permeability followed by decreased mitochondrial viability (8, 20–22). Furthermore, the inhibition of oxidative phosphorylation and inefficacious Na^+^/K^+^ pump function also leads to the accumulation of mitochondrial cytosolic calcium (Ca^2+^). This in turn not only inhibits ATP generation, but also increases the generation of both, reactive oxygen species (ROS) such as hydrogen peroxide (H_2_O_2_) and superoxide as well as, proteolytic enzymes such as phospholipase A2 (10, 19). At this stage, the damaging effects of ROS and proteolytic enzymes are confined within the internal matrix of the mitochondria due to the mitochondrial permeability transition pores (MPTP) which are initially closed (19, 21) (**Figure 1**). Subsequently, continuous action of oxidative stress on the mitochondrial membrane results in a completely permeable mitochondrial membrane with widened MPTP. Following this, ROS and other cytotoxic enzymes are released from the mitochondria to the cytosolic region of cells thus, initiating pro-inflammatory responses at the cellular level (8, 20–22).

### 2.2 Reperfusion triggers pro-inflammatory events

Restoration of oxygen to hypoxic tissues during reperfusion, further exacerbates the existing pro-inflammatory activities initiated during ischemic stress in the mitochondria. This is driven by four major factors: (i) enhanced ROS production, (ii) overexpression of adhesion molecules, (iii) excessive infiltration of pro-inflammatory immune cells and (iv) damaged microvascular endothelial cells. The influx of molecular oxygen during reperfusion, accelerates the production of ROS (23, 24). Enzymes such as xanthine oxidase (23, 25), Nicotinamide Adenine Dinucleotide Phosphate oxidase (NADPH) (23, 25), monoamine oxidase (23, 26) and cytochrome P450 (23, 26) also mediates the production of ROS in an accelerated manner. As discussed previously, during ischemia, excessive accumulation of Ca^2+^, ROS and proteolytic enzymes in the mitochondria causes widening of MPTP resulting in the release of large amounts of ROS, proteolytic enzymes, and pro-apoptotic enzymes such as cytochrome C into the cytoplasm of cells (24). The release of these molecules and enzymes leads to a cascade of events including deoxyribonucleic acid (DNA) single strand breakage, increased production of intracellular pro-inflammatory chemokines and cytokines and overexpression of cell surface adhesion molecules (e.g., Intracellular Adhesion Molecule-1, ICAM-1; Vascular Cell Adhesion Molecule-1, VCAM-1; E- selection and P-selectin) on the injured endothelial cells (27–29). Spontaneously, the release of danger-associated molecular pattern signals (DAMPs), pro-inflammatory cytokines (e.g., Tumor Necrosis Factor-α, TNF-α; Interleukin-6, IL-6) and chemokines (e.g., C-C Motif Chemokine Ligand 2, CCL2) responsible for the activation of Toll-like receptors (TLR) in ischemic tissues, accelerates the inflammatory responses in IRI (19, 30) (**Figure 1**). Endoplasmic reticulum (ER) stress, characterized by the accumulation of unfolded or misfolded proteins in the endoplasmic reticulum lumen during IRI is another major problem caused by IRI (31, 32). ER stress affects the function of unfolded protein responses (UPRs), resulting in compromised protein synthesis and cell function (29, 31, 32). Aphoristically, persistent IRI, if not treated, initiates a milieu of cell death pathways such as necrosis, apoptosis, autophagy, and necroptosis (33, 34) (**Figure 1**). The long-term persistence of these damaging mechanisms eventually leads to graft dysfunction and rejection (8, 22, 27, 28, 35).

### 2.3 Recruitment of innate immune cells during IRI

Detection of the above-mentioned pro-inflammatory signals and cell surface adhesion molecules by circulating leukocytes leads to increased leukocyte receptor binding affinity, resulting in the migration and infiltration of leukocytes across the endothelium of the injured tissues (29, 30, 35). The mechanism of leukocyte infiltration into injured tissues during IRI comprises of 4 major steps: (i) tethering/rolling, (ii) adhesion, (iii) transmigration/extravasation and (iv) infiltration (36–39). During reperfusion, the overexpression of selectins such as L-selectin, P-selectin and E- selectin, on the surface of blood vessel endothelial cells of transplanted grafts, mediates the tethering and rolling of leukocytes towards the endothelial cell ligands (36). Following that, cells expressing integrins such as lymphocyte function-associated antigen-1 (LFA-1), α4 integrins and β1 integrins, adheres to the adhesion receptor molecules which are abundantly expressed on the surface of injured tissues (37, 38). In tissues with IRI, prominently expressed adhesion molecules (e.g., ICAM-1, VCAM-1) promote migration and adhesion of LFA-1 expressing leukocytes to the injured tissue within the first 30 to 60 minutes after reperfusion (36, 37). Pro-inflammatory chemoattractants and/or chemokines released during ischemia and reperfusion, also attract circulating leukocytes towards injured tissues, thus promoting increased chances of adhesion to the surface of injured tissues. Upon firm and successful adhesion to endothelial cells, leukocytes undergo polarization and diapedesis to successfully infiltrate the injured tissue (36, 38). Following successful leukocyte infiltration/extravasation into the injured organ/tissue, cytotoxic signals in the form of cytokines and chemokines are released into the circulation. The release of these signals drives the maturation of lymph node penetrating tissue resident dendritic cells (DC), resulting in increased activation and migration of leukocytes to the site of inflammation (37, 38).

Neutrophils, being the most abundant leukocyte in the circulatory system, are not only the first LFA-1 expressing cells to migrate, adhere and infiltrate the injured tissue but also are key players in augmenting IRI inflammation. Infiltration of neutrophils accelerates the production of pro-inflammatory cytotoxic mediators such as ROS, myeloperoxidases, and Interleukin-17 (IL-17) cytokines (40, 41). Besides neutrophils, activation of C-C chemokine receptor type 2 (CCR2) and CXC3 chemokine receptor 1 (CX3CR1) in IRI promotes the migration, adhesion, and infiltration of pro-inflammatory type M1 macrophages at the injured site (41, 42). Infiltration of M1 macrophages, results in the release of inflammatory-promoting molecules such as IL-6, TNF-α, nitric oxide (NO) leading to continuous inflammatory responses (41, 42). Conversely, the ability of pro-inflammatory M1 macrophages in differentiating into anti-inflammatory M2 macrophages, resulting in the reduction of IRI induced inflammation and recovery of renal function in IRI experimental models were observed (43). However, the exact mechanism responsible for the “switch” remains unknown and must be investigated before macrophages can be considered as potential therapeutic agents along with Tregs for the attenuation of IRI induced inflammation. Furthermore, maturation of resident monocytes (macrophage and dendritic cell precursors) has also been observed in kidneys with IRI. Under inflammatory conditions, “resident” CCR2^−^CX3CR1^high^GR-1^−^Ly6C^−^ monocytes are differentiated into CCR2^+^CX3CR1^low^GR-1^+^Ly6C^high^ pro-inflammatory macrophages and DCs (44, 45). Therefore, the reduction and/or inhibition of pro-inflammatory mediators might offer a potential strategy for the treatment of IRI by reducing the infiltration of pro-inflammatory immune cells.

### 2.4 IRI augments alloimmunity *via* adaptive cell infiltration

Inflammatory responses leading to the onset of graft dysfunction and rejection in the presence of alloantigens are major challenges in organ/composite tissue transplantation. Upon implantation of the donor graft into recipient, allograft immune recognition is initiated by both, preformed alloantibodies, if any, and recipient T-cell receptor engagements. Generally, T-cell recognition occurs *via* 3 pathways: direct pathway, indirect pathway, and semi-indirect pathway (12, 13). The direct allorecognition pathway involves the activation of recipient T cells by peptides presented on donor Major Histocompatibility Complex (MHC) of donor antigen presenting cells (APCs) whereas, the indirect allorecognition pathway involves recipient T cell recognition of donor peptides presented by recipient MHCs on recipient APCs (12, 13). The semi-direct allorecognition pathway involves a hybrid of the two with the recipient T cell recognizing donor MHC peptides presented by donor MHC but on recipient APCs (12, 13). Apart from donor MHC and MHC peptides, other antigenic moieties generated during IRI, such as damaged DNA strands, danger associated molecular pattern (DAMPs) like Heat-Shock Proteins (HSP) and released cytotoxic molecules from dying cells, are presented to the recipient T cells by donor APCs thus activating the direct allorecognition pathway (11, 39). Concurrently, the signals and products released during cell death are also presented to recipient T cells *via* the indirect and semi-direct pathway. As a consequence, upon successful implantation of allografts, the presence of both allorecognition induced inflammation and IRI induced inflammation magnify the allograft infiltration rate of CD4^+^ and CD8^+^ T cells as well as inflammatory macrophages, resulting in the release of abundant pro-inflammatory interleukin cytokines (e.g., IL-6, IL-1, IL-17a) and chemokines from the CXC and CC groups (e.g., CXCL1, CXCL2, CCL2) (11). Additionally, increase of thrombin expression in IRI allografts leads to the increase of CCL2 and CCL3 expression, resulting in increased infiltration of CD4^+^ T cells and decreased infiltration of existing endogenous Tregs (46). The heavy influx of inflammatory events due to increased production of inflammatory mediators, presence of alloantigens, and inflammatory cell infiltration further exacerbates the severity of inflammatory responses whilst compromising the viability of the transplanted allograft.

## 3 Therapeutic potential of Tregs against ischemia and reperfusion injury

Numerous studies have shown the involvement of Tregs in driving a “self-regulatory defense” mechanism following the onset of inflammation (47) however, the exact anti-inflammatory mechanism exerted by Tregs in mitigating ischemic reperfusion injury remain to be elucidated. Tregs are phenotypically heterogenous and are classified into various subsets such as natural Tregs (thymus derived Tregs), inducible Tregs (iTregs), CD8+ Tregs, IL-17 producing Tregs, co-stimulatory Tregs (ICOS+ Tregs), and Type 1 regulatory T (Tr1) cells. Despite the phenotypic difference, these Tregs subsets possess similar characteristics such as FOXP3+ expression, IL-2 cytokine dependency for survival and production of anti-inflammatory cytokine such as IL-10 (48), except for Tr1 cells, which secret high IL-10 but do not express Foxp3 (49, 50). CD4^+^ CD25+ Foxp3 Tregs (both nTregs and iTregs) have been mostly investigated regarding their role in modulating and mitigating the harmful effects of inflammatory immune responses (47, 51); findings from these studies will be highlighted in the following sections.

The efficacy of Tregs in promoting graft (e.g., kidney, heart, liver and vascularized composite tissue) protection and tolerance against the detrimental effects of IRI induced inflammation, besides maintaining immune homeostasis has been well evidenced in numerous *in vitro* and *in vivo* studies (51–54). Jun et al. (2014) showed that, at 72 hours post-reperfusion, the population of circulating endogenous Tregs significantly increased and the majority of Tregs accumulated at injured kidneys. Moreover, the accumulation of Tregs at injured kidneys led to the declination of blood urea nitrogen (BUN) and serum creatinine (sCr) values (55), suggesting the presence of both kidney functional recovery and a pro-reparative mechanism. On the contrary, deterioration of kidney function was observed in experimental groups which were treated with PC61 (anti-CD25 monoclonal antibody) to deplete the presence of endogenous Tregs (55, 56). Treg-mediated immunoregulatory activity was also observed when *ex vivo* autologous expanded Tregs were infused in hepatic IRI experimental models. The levels of serum alanine aminotransferase, aspartate aminotransferase, Interferon-γ (IFN-γ) and IL-17 were significantly reduced in IRI-induced livers receiving Tregs, as compared to non-treated control groups (53). Similarly, Kinsey et al. (55), demonstrated the presence of IL-10 overexpression on Tregs which co-localized at IRI kidneys when, *ex vivo* expanded autologous Tregs were administered prior to the onset of IRI (56).

**Figure 2:**
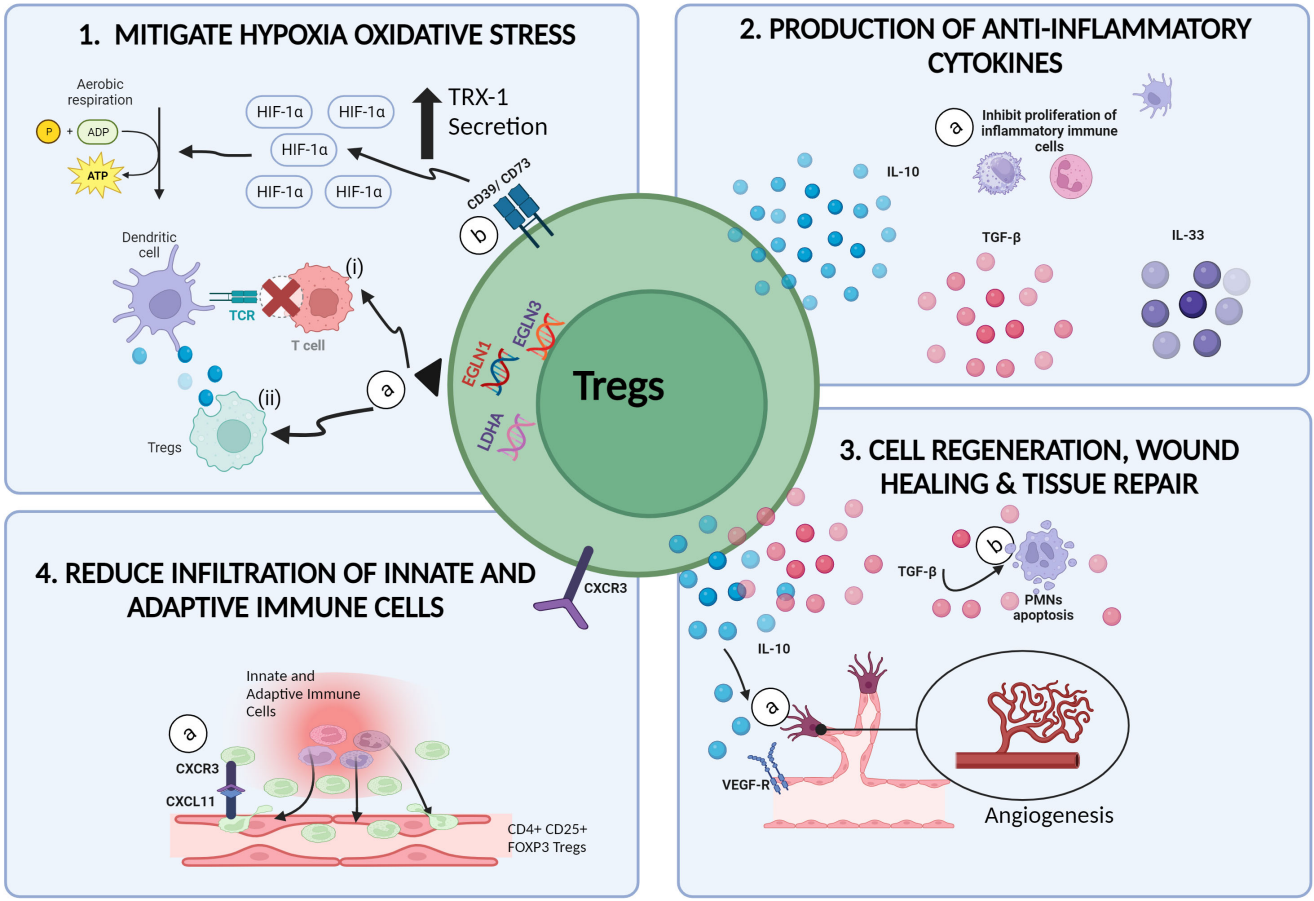
Therapeutic potential of Tregs in mitigating IRI. (1a) In hypoxic microenvironments, the upregulation of EGLN1, EGLN3 and LDHA genes in Tregs, (i) inhibits the communication between alloantigen loaded DCs with naïve T cells, hence preventing the onset of alloimmune immunogenicity induced inflammation. (ii) Tregs are also able to extract and ingest cellular materials from DCs in a process called trogocytosis, resulting in reduced MHCII expression of DCs. (1b) CD39/CD73 expressing Tregs promotes the upregulation of H1F1A expression, resulting in the switch from anaerobic glycolysis to aerobic glycolysis and reduced ROS production. (2a) The secretion of anti-inflammatory cytokines such as IL-10, TGF-β and IL-35 by Tregs promotes the inactivation and growth of inflammatory immune cells such as NKs, DCs and PMNs. (3a) Tregs promote angiogenesis by activating VEGFR-2 receptors via the secretion of IL-10 cytokines. (3b) Tregs are also capable of promoting wound healing and tissue repair in injured tissues by inducing apoptosis and cell death among PMNs via the production of TGF-β. (4a) Increased expression of CXCR3 on Tregs allows the binding of Tregs to the CXCL11 ligands on injured tissue, thus reducing the infiltration of other inflammatory cells (created with BioRender.com).

Findings from the above studies, displayed the involvement of Tregs in attenuating and/or reducing the severity of IRI induced inflammation possibly, by four ways: (i) mitigating cellular oxidative stress, (ii) initiating healing, tissue repair and recovery mechanisms, (iii) preventing and/or reducing the adhesion and infiltration of both, innate immune cells, and adaptive T cells at the injured tissue and (iv) producing anti-inflammatory cytokines (**Figure 2**). Research progress in transplant immunology and autoimmune diseases, has confirmed the immunosuppressive and anti-inflammatory potential of Tregs though, the exact role and mechanisms exerted by Tregs in inhibiting and/or diminishing the progression of IRI induced inflammatory activity remains to be elucidated (55, 56).

### 3.1 Tregs mitigate cellular oxidative stress

The initiation and progression of hypoxia induced damage in tissues deprived of nutrients and oxygen during ischemia, is driven by increased toxic metabolite production (57), oxidative stress (8, 57), mitochondrial dysfunction (8, 22) and overexpression of pro-inflammatory signals released during cell death mechanisms. The continuous cycle of pro-inflammatory events eventually results in allograft rejection and/or organ dysfunction (8, 22, 57). Findings from research on cancer immunotherapies had provided insights on the superiority of Tregs as opposed to other immune cells, in being tolerant and resistant to hypoxia induced stress (58, 59). In hypoxic microenvironments, which is a common scenario in cancer cases, the upregulation of genes such as Egl-9 Family Hypoxia Inducible Factor 1 (EGLN1), Egl-9 Family Hypoxia Inducible Factor 3 (EGLN3) and Lactate Dehydrogenase A (LDHA) in Tregs, represses the antigen presenting efficacy of type -2 conventional dendritic cells (cDC2) thus, inducing immune tolerance and homeostasis (59). The antigen presenting efficiency of DCs can be compromised by Tregs *via* several pathways. Firstly, Tregs can inhibit the communication between alloantigen loaded DCs with naïve T cells, hence preventing the onset of alloimmune immunogenicity induced inflammation (60). Secondly, Tregs are able to extract and ingest cellular materials from DCs in a process called trogocytosis, resulting in reduced MHCII expression of DCs (61, 62) (**Figure 2**). Furthermore, the generation of ROS were shown to stimulate the expression of Programmed Death-Ligand 1 (PD-L1) proteins however the mechanism responsible for this correlation remains vague (63). Hypoxia induced upregulation of PD-L1 expressing DCs, were previously shown to induce differentiation of naïve T cells to Tregs (63), increase the production of chemokines responsible for the infiltration of Tregs (e.g., CXCL5, CCL20) (64) and increase the proliferation of CD169+ macrophages responsible for the recruitment of Tregs at the site of inflammation (59). In a nutshell, the hypoxic resistant characteristics of Tregs, allows Tregs to hit two birds with a stone: (i) thriving throughout ischemia whilst (ii) promoting anti-inflammatory activities during ischemia.

As opposed to most T cells, Tregs are dependent on fatty acid oxidation for persistence and functionality. Depletion of glucose during ischemia promotes proliferation of Tregs at high speed due to the presence of lipid in the circulation. As a result, the increased number of Tregs in the circulation would enable the therapeutic and protective effect of Tregs on the ischemic organ. Interestingly, the inhibition of glycolysis was shown to induce the differentiation of T cells to Tregs due to the increased presence of HIF-1α (65). Therefore, taking into account, both the hypoxic resistant characteristics of Tregs and the presence of Treg-favoring mechanisms during hypoxic oxidative stress, a strategy to manipulate and use these mechanisms to induce Treg mediated immune tolerance may hold the key for the treatment of IRI.

As discussed previously, the overproduction of lactic acid during anaerobic metabolism, impairs the Na^+^-K^+^ pump function, leading to the accumulation of Ca^2+^ and increased ROS production in the mitochondrial matrix (8, 20, 22). The continuous production of ROS during the ischemic episode, reperfusion episode and inflammatory responses in IRI is a major concern as the overexpression of ROS, irreversibly damages the mitochondria, cells, and can even lead to the onset of cancer (58, 64). As compared to other immune cells, the ability of Tregs to withstand the damaging effects of oxidative stress makes them a potential therapeutic option for the treatment and/or management of allografts with IRI. In the presence of oxidative stress, Tregs are protected from undergoing apoptosis due to the ability in producing and secreting high concentrations of thioredoxin-1 (Trx-1) (**Figure 2**). Trx-1 is a cysteine-containing redox-active protein which can scavenge H_2_O_2_, resulting in reduced production and release of oxidative-stress harmful by-products (58, 66, 67).

CD39/CD73 expressing Tregs have been shown to display enhanced tolerogenicity against oxidative stress whilst inhibiting inflammation, in the presence of hypoxia-inducible factor 1-alpha (HIF1A) (68) (**Table 1**). Previous studies have demonstrated both, the upregulation of HIFIA under hypoxia and the protective role of HIF1A on kidneys (68, 80). Since one of the major causes of IRI is related to the presence of oxygen deprivation, utilizing the benefits which HIF1A can exert on kidneys during this episode would help in the effort to reduce the harmful effects of IRI in kidney allografts post transplantation. Recently, HIFIA were shown to protect cardiac cells against IRI induced inflammation by modulating mitochondrial metabolism and promoting angiogenesis (20). Jarvis et al. (68) had developed an optimized protocol for the expansion of Tregs with significant expression of CD39 and CD73 markers. The administration of CD73 expressing Tregs in recipients with kidney IRI, led to the upregulation of H1F1A expression in kidney cells, resulting in the switch of anaerobic glycolysis to aerobic glycolysis (68). In the context of IRI, this metabolic switch would result in the reduction of lactic acid production whilst preventing damages to the mitochondria and cells. Although this approach seems promising, it is crucial to determine the “right amount/optimal amount” of HIF1A-CD73 expressing Tregs interaction for the induction of immune tolerance as this hypoxic factor can also contribute to the onset of cancer.

**Table 1:** Therapeutic role of Tregs in mitigating IRI induced inflammation.

	Tregs activity	Experimental model	Reference(s)
**Oxidative stress**	CD4+ CD25^high^ Tregs demonstrated antioxidative capacity and maintained immunosuppressive properties despite being exposed ROS such as H_2_O_2_	*In vitro*	(57)
	Mtor pathway inhibition during ischemia/nutrient starvation promotes the transcription of FOXP3 and proliferation of Tregs. As a result, FOXP3 Tregs are reprogrammed to adapt to energy demands by enhanced oxidative phosphorylation and resist apoptosis	*In vitro*	(69)
**Production of anti-inflammatory cytokines**	IL-33 expressing Tregs suppressed the proliferation of CD4+ T cells *via* the secretion of IL-10 and TGF-β	*In vitro*	(70)
	IL-10 inhibits the activity of pro-inflammatory cytokines and innate immune cells such as monocytes, macrophages, and dendritic cells		(71)
	IL-10 expressing Tregs reduced the expression of IFN-γ and IL-17 in experimental models with hepatic IRI	*In vivo*	(53)
**Cell regeneration, wound healing, and tissue repair**	Keratinocyte growth factor (kgf) expressing Tregs promoted the proliferation of lung alveolar epithelial cells	*In vitro; In vivo*	(72)
	IL-10 secreting FOXP3+ Tregs in zebrafishes promoted tissue repair in injured tissues by producing tissue specific regeneration growth factors	*In vitro; In vivo*	(73)
	Tregs suppressed aldosterone-mediated vascular injury, by preventing and/or reducing the infiltration of pro-inflammatory innate and adaptive immune cells	*In vitro; In vivo*	(74)
	Tregs promoted neurogenesis in brain IRI experimental models *via* the upregulation of IL-10	*In vivo*	(75)
	CD4+ CD25+ FOXP3 Tregs which infiltrated into injured muscles tissues and promoted proliferation of muscles cells	*In vitro; In vivo*	(76)
	CCR10-expressing Tregs induced angiogenesis of cancer cells in the presence of hypoxia		(60)
	FOXP3^high^ Helios^high^ Tregs promoted angiogenesis in the bone marrow *via* the vascular endothelial growth factor (VEGF)A/VEGF receptor 2 (VEGFR2) pathway	*In vitro; In vivo*	(59)
	Proliferation and differentiation of hair follicles are stimulated by Jag 1 (ligand of Notch pathway), which is upregulated by skin tissue resident Tregs	*In vitro; In vivo*	(77)
	Tregs promoted angiogenesis and tissue repair in ischemic retina	*In vitro; In vivo*	(78)
	IRI kidneys containing IL-33R^+ and^ IL-2Ra^+^ Tregs had increased expression of pro-angiogenic and cell regeneration genes such as *Nrp1*, *Kdr*, *Mfge8*, *Vegfa*, and *Icam1*	*In vivo*	(79)

Overall, Tregs can counter oxidative stress *via* several routes. As discussed earlier, besides producing high levels of Trx-1, Tregs are also able to limit the expression of γ-glutamylcysteine synthetase and glutathione synthesis which is crucial for the proliferation and activation of pro-inflammatory T cells by DCs (67, 81). Developments in autoimmune disease findings, had also revealed the potency of Tregs in reducing and/or inhibiting the production of harmful oxidative stress by-products. A decrease in serum oxidized low-density lipoprotein (ox-LDL), an oxidative stress product, were observed in Amyotrophic lateral sclerosis patients administered with *ex vivo* expanded autologous Tregs, as compared to the untreated control group (82). It is important to note that, anti-oxidative mechanisms are commonly modulated by antioxidant enzymes rather than endogenous cells itself. Therefore, identifying the exact anti-oxidative enzymes manipulated by Tregs for the suppression of IRI induced oxidative stress would hold the key to treat and/or to prevent oxidative stress in many other diseases, besides graft IRI. To date, *in vivo* data demonstrating the reduction and/or inhibition of IRI induced oxidative stress by Tregs remain insufficient therefore, understanding the route taken by Tregs in reducing oxidative stress may offer a potential strategy to prevent the onset of IRI in allografts instead of treating IRI grafts.

### 3.2 Tregs facilitates cell regeneration, wound healing and tissue repair

Even though the occurrence of inflammatory activity is part of the defense mechanism in tissue injury, excessive and persistent pro-inflammatory activity in IRI grafts can lead to the formation of fibrosis and scar tissues (e.g., heart), acute kidney injury (AKI) (e.g., kidney), graft dysfunction and/or graft failure. The indispensable roles of Tregs in promoting angiogenesis, wound healing, and tissue repair in addition to maintaining immune homeostasis, makes them an excellent candidate to be exploited for the treatment of grafts with IRI. To date, the cellular and molecular pro-reparative mechanisms mediated by Tregs in allografts with IRI has not been fully elucidated albeit, evidenced in numerous studies (**Table 1**). Tregs were reported to facilitate skin healing and wound repair by decreasing both, the expression of IFN-γ and pro-inflammatory macrophage accumulation at injured tissues *via* the Epidermal Growth Factor Receptor (EGFR) pathway (83). Wang et al. (75), demonstrated the ability of Tregs in promoting neurogenesis in brain IRI models *via* the upregulation of IL-10 (75).

The formation of new blood vessels, a process known as angiogenesis, plays an important role in tissue repair and wound healing (**Figure 2**). Generally, Tregs mediated pro-angiogenesis activity are driven by (i) increased production of anti-inflammatory cytokines, (ii) upregulation of angiogenesis promoting genes and proteins and (iii) activation of growth factor receptors to produce growth factors (75, 79, 84). Secretion of IL-10 anti-inflammatory cytokines and activation of Vascular Endothelial Growth Factor Receptor-2 (VEGFR-2) for the production of VEGFs (85) by Tregs in renal cell carcinoma, were shown to promote aggressive angiogenesis resulting in cancer cell expansion and metastasis (84, 86, 87). Considering this, the secretion of pro-angiogenic stimulators such as IL-10 and VEGF in IRI grafts, may promote angiogenesis which would eventually result in cell regeneration and tissue repair. The upregulation of pro-angiogenic and cell regeneration genes (e.g., *Nrp1*, *Kdr*, *Mfge8*, *Vegfa*, *Icam1*) by IL33R^+^ and IL-2Ra^+^ expressing Tregs in IRI kidneys, demonstrated by do Valle Duraes et al. (79) supported the above-mentioned hypothesis (79).

Nonetheless, Tregs were also shown to negatively regulate angiogenesis in certain tissues and conditions (84, 88, 89). Tregs inhibited endothelial cell angiogenesis by inducing the downregulation of VEGF and upregulation of the pro-apoptotic Notch signaling pathway (88). The administration of exogenous Tregs in CD28 Knock out (KO) mice with hindlimb IRI resulted in reduced neovascularization rate following IRI (89). Despite the numerous evidence of angiogenesis inhibition by Tregs, it is noteworthy that dysfunctional Tregs, rather than functional Tregs were reported to be involved in ischemia induced inflammation (90). In ischemic cardiomyopathy, excessive proliferation of immunomodulatory lacking- dysfunctional Tregs led to the overexpression of pro-inflammatory cytokines, anti-angiogenic activity, and formation of fibrosis (90). Adoptive transfer of functional Tregs to replace the dysfunctional Tregs in the context of ischemic cardiomyopathy may be a fruitful approach to overcome the complications in ischemic cardiomyopathy cases.

Tregs are also able to inhibit and/or manipulate the behavior of certain pro-inflammatory innate immune cells to promote tissue repair (91). In the presence of activated Tregs, polymorphonuclear neutrophils (PMNs) decreased the production of IL-6 pro-inflammatory cytokines and increased the production of anti-inflammatory molecules like IL-10, TGF-β1, heme oxygenase-1 (HO-1) and the suppressor of cytokine signaling 3 molecule (SOCS3) (91). Moreover, activated Tregs were also able to promote accelerated wound healing and tissue repair in injured lungs by inducing apoptosis and cell death among PMNs *via* the production of TGF-β (92). Aside from neutrophils, Tregs are also able to manipulate and reduce the pro-inflammatory activity exerted by other innate immune cells such as macrophages. Tregs promoted anti-inflammatory activity in cardiac tissues and inhibited the formation of scar tissues by preventing extracellular collagen degradation, as well as increasing the secretion of cytokines responsible for the proliferation of anti-inflammatory M2 macrophages (59, 93).

Owing to this finding, additional and detailed studies to confirm the exact mechanisms and/or trigger factors responsible for Treg mediated angiogenesis and anti-angiogenesis is warranted before these cells can be used as a therapeutic option in IRI.

### 3.3 Tregs secretes anti-inflammatory cytokines

The secretion of anti-inflammatory cytokines such as Interleukin-10 (IL-10) (56, 94–96), Interleukin-35 (IL-35) (96), Interleukin-4 (IL-4) (97, 98) and Transforming growth factor-beta (TGF-β) (96) by Tregs are the few major drivers responsible for the induction of Treg-mediated IRI inflammatory suppression (**Figure 2**) (**Table 1**). The attenuation of IRI inflammation observed in Recombination Activation Gene-1 (RAG-1) KO mice administered with Tregs as opposed to IL-10 KO Tregs (56), emphasized the importance of IL-10 in Tregs for the dampening of aberrant hypoxia induced inflammatory response. IL-10 exerts immunosuppressive effects by inhibiting the pro-inflammatory activity of macrophages and monocytes *via* the JAK/STAT signaling pathway, stimulating the production of anti-inflammatory molecules such as Interleukin-1 receptor antagonist (IL-1RA), soluble Tumor necrosis factor-alpha (TNF-α) and Interleukin- 27 (IL-27) as well as enhancing the function of Tregs (99). TGF-β on the other hand, is crucial for the differentiation of naïve T cells into Tregs, regulation of FOXP3^+^ expression and maintenance of functional Tregs *via* the IL-2/STAT5 signaling pathway (100, 101). IL-10 and TGF-β expressing Tregs also induces immune tolerance by decreasing the expression of pro-inflammatory cytokines, CC and CXC chemokines as well as adhesion molecules (56, 94–96, 99, 102). Consequently, the proliferation and activation of innate immune cells namely natural killer (NK) cells, granulocytes, DCs, monocytes, effector T cells and B cells are hampered. IL-4 was previously shown to exert both, negative and positive effect against Tregs. On a positive note, IL-4 are able to enhance the immunosuppressive activity and prolong the survival of Tregs (97, 98). Yang et al. (2017), showed the ability of IL-4 stimulated Tregs in inducing immune suppression *via* the secretion of granzymes. On the contrary, IL-4 and granzyme production also prolongs inflammatory events by inducing apoptosis among cytotoxic lymphocytes (103) which would eventually compromise the function of the transplanted graft.

### 3.4 Tregs reduces the infiltration of pro-inflammatory immune cells

Tregs are also able to decrease the progression of IRI induced inflammatory responses and induce immune tolerance by adhering to the adhesion molecules expressed on injured endothelial cells henceforth, reducing the infiltration of pro-inflammatory leukocytes (**Figure 2**). Overexpression of G-protein-coupled receptor 9 (CD183), commonly known as C-X-C motif chemokine receptor 3 (CXCR3) on Tregs were shown to be responsible in promoting Tregs mediated anti-inflammatory responses in IRI kidneys (52, 104, 105). Overexpression of CXCR3 on Tregs, were shown to increase both, rate of migration and binding and/or adhesion probabilities of Tregs to the CXCL11 ligand at injured tissues as compared to other pro-inflammatory leukocytes henceforward, reducing the severity of IRI inflammation (104, 105). Increased expression of CXCR3 on Tregs also led to the overexpression of anti-inflammatory cytokines (e.g., Interleukin-10), antioxidants (e.g., Superoxide dismutase, SOD and glutathione peroxidase, GSH-Px) and downregulated the expression of pro-inflammatory cytokines (e.g., IL-6 and TNF-α) in experimental models with renal IRI (105). However, although IRI induced inflammation can be attenuated or reduced by the binding of CXCR3 expressing Tregs to CXCL11 ligand, it is important to note that the binding of CXCR3 to other ligands such as CXCL9 and CXCL10 would increase the recruitment and infiltration rate of neutrophils and natural killer T cells, resulting in the worsening of pro-inflammatory events (52, 105, 106). Therefore, inhibition of these pro-inflammatory ligands and administration of CXCR3 rich Tregs in IRI renal grafts may promote tolerance against IRI induced inflammation. Another possible mechanism utilized by Tregs in reducing IRI induced inflammation is by binding to a class IV semaphorin known as, Semaphorin 4A (Sema4A) (107). The binding of neuropilin-1 (Nrp-1) on Tregs to Sema4A before the onset of IRI, resulted in accelerated expansion of endogenous Tregs which in turn, decreased the infiltration rate of innate immune cells such as macrophages and neutrophils into injured tissues leading to the alleviation of renal IRI inflammation (56, 107).

## 4 Treg based cellular therapy: challenges and stratagems to combat ischemia and reperfusion injury

The fundamental role played by Tregs in promoting immune tolerance, controlling pro-inflammatory activities, and maintaining immune homeostasis in both, alloantigen immunogenicity and inflammation has been well evidenced (52, 55, 56). The significant increase of endogenous Tregs proliferation and enhanced trafficking of Tregs at the site of inflammation, resulting in improved allograft function post IRI, demonstrated an obvious anti-inflammatory “antagonistic” relationship between Tregs against IRI progression. Regardless of the promising existence of Treg-mediated anti-inflammatory activity in allografts against IRI induced inflammation and alloantigen immunogenicity, depending completely on “endogenous” Tregs alone, seems unreasonable due to a number of reasons. Firstly, the number of endogenous Tregs in the circulation to drive anti-inflammatory responses is insufficient, as they only make up 5-10% of the total CD4^+^ T cell population (12, 108). Secondly, since Tregs in the circulation exists in small numbers, the time taken for the proliferation of Tregs to reach optimal levels before, initiating an anti-inflammatory response, might be too long. Grafts with IRI, will be severely damaged due to the co-existence of several pro-inflammatory events taking place simultaneously against the graft (108). Thirdly, the number of circulating endogenous Tregs are too little to compete, reduce and/or to prevent the adhesion and infiltration of other inflammatory driving leukocytes such as DCs, macrophages, granulocytes, and T cells at organs with IRI. Taking into consideration of the above-mentioned limitations of endogenous Tregs- feasible, reasonable, and strategic approaches to (i) increase the number of Tregs in the circulation, (ii) to enhance the survival of Tregs whilst, (iii) ensuring targeted delivery of Tregs are necessary to prevent, reduce and/or to alleviate IRI induced inflammation in allografts.

### 4.1 Adoptive transfer of *ex-vivo* expanded Tregs

Adoptive transfer of Tregs is a direct, convenient, and promising therapeutic strategy to increase the number of Tregs in the circulation for the prevention, inhibition and/or alleviation of IRI induced inflammatory responses in transplanted grafts. Several *in vitro*, *in vivo*, pre-clinical and clinical studies had demonstrated the efficacy of adoptive Treg cellular therapy for the treatment of autoimmune disorders (12, 109) and induction of immune tolerance in organ transplantation (12, 110). AKI was prevented in kidney grafts of recipients who received an adoptive transfer of polyclonally expanded, autologous Tregs before the onset of IRI (55). The kidneys of lymphocyte deficient RAG-1 KO mice were protected from IRI when polyclonally expanded, autologous Tregs were infused 2 weeks prior to the onset of IRI (56). Tregs reduced and/or prevented the progression of IRI-induced pro-inflammatory responses in RAG-1 KO mice kidneys by producing IL-10 cytokines. Despite understanding the therapeutic effect of IL-10 producing Tregs in IRI, it is noteworthy that the absence of both, endogenous T cells and B cells in RAG-1 KO mice, does not display the precise effect of IL-10 producing Tregs in treating IRI. Besides IL-10, TGF-β producing Tregs were reported to suppress and/or to prevent IRI-induced inflammation of the liver, when administered into C57BL/6 mice 24 hours before the onset of IRI (111). Gandolfo et al. (112) on the other hand, demonstrated increased trafficking of Tregs at the interstitium of IRI kidney outer medulla, when polyclonally expanded autologous Tregs were infused 24 hours after IRI. The heavy trafficking of Tregs at the site of injury, prevented and/or reduced the infiltration percentage of pro-inflammatory innate leukocytes such as CD11c+ F4/80 DCs as well as lymphoid T cells (112). As a result, abundant anti-inflammatory cytokines released by activated Tregs suppresses the rate of IRI-induced pro-inflammatory responses hence, resulting in a lesser extend of kidney damage as well as promoting kidney recovery (112). Overall, it can be presumed that adoptive cellular transfer of Tregs before and/or after the onset of IRI, may promote protection against transplanted grafts by ameliorating IRI-induced inflammatory effects. Although polyclonally expanded Tregs significantly demonstrated protection of renal grafts against the detrimental effects of IRI, the functional stability and increased probability of polyclonal Tregs in exhibiting pan-immunosuppression against broad spectrum of unspecific targets remain a concern (47, 113).

The existing limitation of using polyclonally expanded Tregs, although convenient, led to the discovery and development of donor antigen specific Tregs (ds-Tregs). In transplanted grafts, pro-inflammatory events are triggered by both, alloantigen recognition and IRI. In addition to that, IRI-induced inflammatory responses have also been shown to augment the severity of alloimmune response, resulting in graft rejection, and/or graft failure. As opposed to polyclonal Tregs, ds-Tregs offer a more potent and targeted approach in controlling inflammation and inducing immune tolerance since these cells can migrate and act specifically on the donor tissue containing the recognized alloantigens (12, 113). We hypothesize that, by utilizing ds-Tregs, the pro-inflammatory responses induced by allo-antigen recognition could be controlled and/or alleviated hence, indirectly suppressing oxidative stress, ROS generation and cell damaging mechanisms exerted on the transplanted graft, in addition to IRI. The anti-inflammatory and immunomodulatory potential of ds-Tregs in SOT has been extensively studied however, to the best of our knowledge, the potency of ds-Tregs for the prevention and/or treatment of IRI are yet to be discovered. Despite the efficacy of ds-Tregs in promoting immune tolerance, the purity and yield of expanded ds-Tregs remains a concern (12, 47, 114). Administration of low purity ds-tregs into transplant patients, may further aggravate the existing inflammation due to the presence of non-Tregs pro-inflammatory cells. Recently, Mathew et al. (114), established a protocol for the expansion of FOXP3 rich, immunosuppressive ds-tregs by using recipient Tregs stimulated with donor B cells. The expanded ds-tregs significantly, inhibited the proliferation of recipient T cells at a Tregs to responder cell ratio of (1:10) to (1:250) (114). Furthermore, as opposed to non-specific polyclonal Tregs, only a small number of ds-tregs are required to induce immunosuppression (113).

### 4.2 Endogenous Tregs proliferative stimulants

Alternatively, the immunomodulatory benefits of Tregs can also be exploited indirectly for the prevention and/or treatment of IRI by using non-cell-based therapeutic approaches. Cytokines such as TGF-β and Interleukin -2 (IL-2), play a crucial role in the survival, development, and immunosuppressive potential of Tregs (12, 115). The transcriptional identity of Tregs, characterized by the presence of CD25^high^ FOXP3^high^ is upregulated by Signal Transducer and Activator of Transcription 5 (STAT5) activation under the influence of IL-2 expression (115). As compared to other pro-inflammatory leukocytes such as T cells and NKs, CD25 proteins are significantly overexpressed on Tregs. Therefore, administration of IL-2, even in low doses, promotes selective targeting of IL-2 on CD25 receptors which are abundantly expressed on Tregs (115–117), resulting in the survival and proliferation of highly immunosuppressive CD25^high^ FOXP3^high^ Tregs. Owing to this theory, administration of low dose IL-2 along with *ex vivo* expanded autologous Tregs, may enhance the proliferation and prolong the survival of both, endogenous and infused Tregs in allografts with IRI. Additionally, inhibition of thrombin expression on injured endothelium, were shown to enhance the accumulation of both, endogenous and *ex-vivo* infused Tregs as well as M2 anti-inflammatory macrophages at the site of inflammation (46). Furthermore, recent studies in the application of normothermic and/or hypothermic machine perfusion of donor organs prior to transplant have shown promising results in reducing the extent of IRI induced damage on allografts (118). It is reasonable to speculate that lesser extent of IRI damage on the allograft would enhance the accumulation of Tregs at the injured site in a shorter period resulting in fast recovery. Enhanced migration, accumulation, and infiltration of Tregs at the site of inflammation would reduce and/or prevent both, the infiltration of other non-Tregs pro-inflammatory leukocytes and production of pro-inflammatory cytokines (117). The secretion of anti-inflammatory cytokines by Tregs at the site of inflammation would eventually aid in the alleviation of IRI induced inflammatory responses and accelerated healing of the injured graft.

Immunosuppressive (IS) agents, from the family of steroids, calcineurin inhibitors and mTOR inhibitors are routinely administered in transplantation to prevent the onset of aggressive inflammatory responses (116, 119). Interestingly, mTOR inhibitor IS drugs were found to selectively promote the proliferation, survival, and enhancement of suppressive phenotypes on Tregs whilst preventing the proliferation of effector T cells (116, 120). Therefore, the administration of mTOR inhibitor IS drugs such as Rapamycin, Sirolimus (121) and Everolimus (120) in SOT/VCA alone or along with *ex vivo* expanded autologous Tregs, preferably ds-Tregs, may prevent and/or alleviate the detrimental effects of IRI against grafts. While the effects of IS drugs from the mTOR inhibitor family on Tregs are extremely promising, the line which differentiates the “yin effect” from the “yan effect” of mTOR inhibitors against Tregs must be investigated in detail since precise mechanisms underlying mTOR inhibitor tolerogenic activity remains vague. The involvement of mTOR inhibitors in the progression of cancer, is one of the major reasons these IS drugs, are not the first choice of option during transplantation (122). In addition to this, IS drugs have also been reported to affect the expression of FOXP3 in Tregs therefore, the optimal dosage for optimal Tregs immunosuppression induction must be understood before they could be applied in the clinical setting. Alternatively, other compounds such as N,N-dimethylsphingosine (DMS) (123, 124), Histone deacetylase inhibitors (125, 126), HMG-CoA reductase inhibitors (127) and Sphingosine-1-phosphate receptor agonists (128) were also reported to protect organs against the detrimental effects of IRI by increasing the proliferation rate of immunosuppressive Tregs however, more detailed investigations on the exact underlying mechanisms of the above-mentioned molecules should be understood.

### 4.3 Localized administration route

To the best of our knowledge, the route used for the adoptive transfer of *ex vivo* expanded Tregs in the majority of current experimental models and clinical trials are *via* the convenient and minimally invasive intravenous (IV) route (129, 130). The route used for the delivery of therapeutic agents in general, plays a huge role in determining the effectiveness of a therapy. Previously, it was shown that the majority of IV administered therapeutic Mesenchymal Stem Cells (MSCs) were trapped within the pulmonary capillaries thus preventing optimal distribution to the targeted organs (131). On the contrary, IV-infused MSCs in recipients treated with sodium nitroprusside vasodilators, were able to reach the targeted organs without being trapped in the lungs, suggesting that the entrapment of MSCs could be due to their large size (129, 130, 132, 133). IV infusion of actively proliferating *ex vivo* expanded Tregs, may undergo the same problem resulting in lesser accumulation of Tregs at the IRI organ. In addition to size, other factors such as cellular adhesion to the vascular endothelium may contribute to the incidence of pulmonary cell trapping (133). In support of this, Lyu et al. (134), demonstrated the presence of CD49d lung homing marker on umbilical cord blood derived Tregs which resulted in the accumulation of Tregs at the lungs, post infusion (134). IV infused cells were also found to be distributed to other organs such as liver and spleen, after by-passing the lungs resulting, in increased cell loss and lesser accumulation at the targeted organ (129, 131). This limitation would result in the inability of Tregs in suppressing and/or alleviating IRI induced inflammation due to lesser number of Tregs accumulation at the site of inflammation (130). In order to overcome this problem, an alternative route is required to enhance the delivery of Tregs at the targeted organ. Direct injection of Tregs on grafts with IRI may offer a more promising outcome although, this approach may introduce more injury to the stressed organ/tissue (130).

### 4.4 Targeted delivery of Tregs using nanotechnology

Developing efficient cell delivery methods using nanomaterials could also significantly improve the effective implementation and outcomes of Treg-based cell therapy in IRI. The attachment of nanomaterials on the extracellular surface of Tregs is an approach which could be investigated for the treatment of IRI. As discussed earlier, the overexpression of adhesion molecules such as ICAM-1, VCAM-1 and MAdCAM-1 on the surface of IRI grafts following reperfusion, initiates the migration and infiltration of pro-inflammatory leukocytes at the site of injury (36–38), triggering a cascade of pro-inflammatory responses. In order to prevent this, the coating and/or binding of Tregs to nanoparticles which could selectively bind to adhesion molecules commonly upregulated in IRI, would reduce the infiltration of pro-inflammatory leukocytes at the site of inflammation. The usage of nanoparticles to aid and/or to enhance the efficacy of Treg based cellular therapy however warrants in-depth investigation to assess the costs, accuracy, and toxicity of nanoparticles against Tregs as well as other organs in the system.

## 5 Conclusion

Cellular adoptive therapy using Tregs for the induction of immune tolerance are a preferred choice as compared to synthetic drugs, since they are naturally existent and are non-toxic to our system. Although the advancements in immunology-based research have enabled us to isolate, identify and understand some of the pathways utilized by Tregs in alleviating and/or reducing the detrimental effects of IRI induced inflammatory events, all that glitters are not gold. One of the major challenges faced in the utilization of Tregs as a therapeutic option, is the ability of culturing and expanding *ex vivo* Tregs-expressing markers such CD73 which aids in the induction of immune tolerance, specifically in an IRI setting. Furthermore, it has been brought to attention that, a subset of FOXP3^+^ Tregs were able to produce and secrete pro-inflammatory cytokines under certain conditions (135). Therefore, the second challenge in using Tregs as a therapeutic option for the treatment of IRI is the lack of data on the role of certain stimulatory factors responsible for the differentiation and plasticity of Tregs. The ability to overcome at least a few of the challenges reviewed in this review, can open alternative and effective pathways in using Tregs for the treatment of IRI in clinical trials. This review is timely and further investigation is warranted to explore the various methods Tregs could be manipulated for the treatment of IRI.

## Author contributions

SJ: Writing – original draft, Writing – review & editing. JM: Funding acquisition, Supervision, Writing – review & editing. ZZ: Funding acquisition, Supervision, Writing – review & editing, Conceptualization. JL: Writing – review & editing, Supervision, Conceptualization, Validation.
